# Examining the Relationship Between Daily Activity Levels and Elder Abuse and Neglect: Bringing Light on Elder Abuse

**DOI:** 10.1111/jan.17026

**Published:** 2025-05-08

**Authors:** Gül Bülbül Maraş, Enes Ünlübaş

**Affiliations:** ^1^ Elderly Care Program Vocational School of Health Services, İzmir Demokrasi University İzmir Turkey

**Keywords:** daily living activity level, elder abuse, Geriatric Mistreatment Scale, neglect, nurses, nursing, older adults, risk factors, sociodemographic characteristics, Turkey

## Abstract

**Aim:**

To examine the relationship between sociodemographic characteristics, daily activity levels, and elder abuse among older adults in Turkey.

**Design:**

A descriptive, cross‐sectional study was conducted across seven provinces.

**Methods:**

The study was carried out between January and August 2023, involving 448 older adults. Data were collected using the Sociodemographic Form, Mini‐Mental State Examination, Barthel Index, and Geriatric Mistreatment Scale.

**Results:**

Approximately one in four participants reported experiencing at least one form of non‐sexual elder abuse, with psychological abuse being the most common. Sons and male caregivers were frequently identified as perpetrators. While no significant relationship was found between daily activity dependency and abuse, factors such as lower education, poor self‐rated health, intrafamily conflict, and regional variation were significantly associated with increased risk.

**Conclusion:**

Elder abuse remains a prevalent and underrecognized issue, shaped by both individual vulnerabilities and contextual dynamics. The absence of a direct link between functional dependency and abuse suggests the need to consider broader social and environmental factors.

**Implication for the Profession and Patient Care:**

Routine screening for elder abuse, particularly psychological abuse, should be part of geriatric care. Training healthcare professionals to identify risk factors—such as perceived health, family dynamics, and regional differences—and using culturally sensitive, sociodemographic assessments can improve early detection and intervention. Strengthening collaboration and community‐based support is key to ensuring older adults' safety and well‐being.

**Impact:**

The study highlights elder abuse in Turkey as a complex, regionally variable issue influenced by health perception and family dynamics. It calls for targeted, community‐based interventions and stronger collaboration among healthcare providers, nurses, social workers, and policymakers, emphasising the need for improved social security and culturally sensitive training.

**Reporting Method:**

This study followed STROBE guidelines.

**Patient or Public Contribution:**

No patient or public contribution.

**Summary**

- What does this paper contribute to the wider global clinical community?
  ○ It reveals significant regional variations and rates of elder abuse in Turkey, providing guidance for elder care practices that consider cultural differences.
  ○ Identifies family conflict and poor self‐perceived health as key abuse risk factors, emphasising targeted psychosocial screening.
  ○ Questions traditional assumptions linking functional dependency to abuse, prompting a rethink of current elder abuse risk profiles.

## 1 Introduction

The global elderly population is rapidly increasing and is projected to reach 1.4 billion by 2030, 2.1 billion by 2050, and 3.2 billion by 2100 (Naja et al. 2017). In Turkey, the proportion of individuals aged 65 and over has risen from 8.8% to 10.2% over the past 5 years, according to the Turkish Statistical Institute (TURKSTAT 2023). Population projections indicate that this ratio will increase to 12.9% by 2030, 16.3% by 2040, 22.6% by 2060, and 25.6% by 2080 (TURKSTAT 2023). As individuals age, deteriorating health and limited mobility make independent living more challenging, which increases the need for care and dependency (Yon et al. 2017).

The Centers for Disease Control and Prevention (CDC) identifies elder abuse and neglect as a public health problem requiring formal surveillance. At the same time, the World Health Organisation (WHO) prioritises elder abuse and neglect as a key issue in its Decade of Healthy Aging (2021–2030) initiative (CDC 2016; Lewis et al. 2023; WHO 2022). The WHO and the International Network for the Prevention of Elder Abuse (INPEA) define elder abuse as “a single or repeated act, or lack of appropriate action, occurring within any relationship where there is an expectation of trust, which causes harm or distress to an older person” (Naja et al. 2017; WHO 2022). According to this definition, a trust relationship exists between the older adult and the perpetrator, who may be as distant as a salesperson or as close as a family member (Lewis et al. 2023; WHO 2022). Elder abuse involves physical, emotional, or mental mistreatment, harm, and suffering inflicted on the elderly individual (United Nations 2024; WHO 2022). Neglect is defined as the failure, whether intentional or unintentional, to meet the social, physical, and emotional needs of an elderly individual (CDC 2016; WHO 2022; Zhang et al. 2022). Indicators of elder abuse include medical findings that are difficult to explain, such as recurring wounds or injuries and unexplained weight loss (Özmete 2016).

## 2 Background

Abuse and neglect occur across all societies, economic levels, ethnicities, and religions (CDC 2016). The most common places for abuse and neglect include the older adult's home, hospitals, nursing homes, and day‐care centres (Yon et al. 2017). While cultural differences in elder abuse may exist, most cases occur within the family (Pillemer et al. 2016). The traditional family structure, which often conceals family secrets, frequently leads to the neglect of such issues (Dabas and Isikhan 2019; United Nations 2024).

Elder abuse prevalence varies significantly across regions. In the United States, elder abuse is reported at 10%, while in Turkey, it ranges from 13.6% to 32.5% (Çevik et al. 2021; Pak 2020). According to Dong's (2015) study, the rates in Asia range from 14% in India to 36.2% in China and Europe, from 2.2% in Ireland to 61.1% in Croatia (Dong 2015). According to the study by Zhang et al. (2022), the rates in Asia are reported as 50.2% in India, in Europe as 29.3% in Spain, in Africa as 46% in Egypt, and in America as 8.1% in Mexico (Zhang et al. 2022). A meta‐analysis reveals that globally, one in six older adults (15.7%) experienced abuse in the past year (Yon et al. 2017). However, it is believed that the problem of elder abuse and neglect in the community is more widespread than initially thought (WHO 2024).

Each year, millions of older individuals experience functional status loss, decreased quality of life, dependency, helplessness, social isolation, stress, and psychological distress due to abuse and neglect (Çevik et al. 2021; Yılmaz et al. 2022). Elder abuse is influenced by social factors, such as mental health issues, social isolation, and living alone, as well as demographic characteristics, including gender, education level, low income, rural residence, chronic illness, and caregiver traits (CDC 2016; Dong et al. 2013). Older individuals dependent on others to meet their basic needs are particularly at risk for abuse and neglect (WHO 2002). Elder abuse is challenging to detect because victims often do not report it, feeling guilt, shame, fear, or living in social isolation (Rosen et al. 2016; Daşbaş et al. 2019; Pillemer et al. 2016). Additionally, healthcare professionals often lack awareness and face difficulties recognising signs of elder abuse due to factors such as inadequate training, misconceptions, heavy workloads, cultural taboos, and the subtle or nonspecific nature of abuse indicators. Distinguishing these signs from normal aging processes, such as bruising or confusion, is particularly challenging (Daşbaş et al. 2019; Garma 2017; Rosen et al. 2016). This lack of recognition delays intervention and allows abuse to continue unaddressed (WHO 2022).

With the increasing older population, elder abuse has become more frequently discussed in Turkey. The first scientific study on elder abuse in Turkey was conducted on older individuals applying to nursing homes (Artan 1996). Various assessment tools have been used in the literature to identify and evaluate elder abuse. Some tools are designed to generally detect elder abuse (Reis and Nahmiash 1998); some focus on identifying specific types of elder abuse (Conrad et al. 2011; Giraldo‐Rodríguez and Rosas‐Carrasco 2013); others aim to detect the likelihood of elder abuse (Fulmer 2003; Yaffe et al. 2008), and some are targeted at identifying risk factors for elder abuse (Neale et al. 1991; Schofield and Mishra 2003). In studies conducted in Turkey, it is noted that primarily, a single measurement tool has been used to assess the risk of elder abuse (Çevik et al. 2021; Özmete 2016; Pak 2020). Studies on the types of elder abuse are essential for ensuring a safer and healthier life for the aging population (Dong et al. 2013; Daşbaş et al. 2019).

An essential indicator of quality of life in old age is living independently without relying on others (Şahin et al. 2016). Older individuals dependent on others for their basic needs are particularly vulnerable to abuse and neglect (Pillemer et al. 2021; Storey 2020). Independence entails performing basic daily living activities and fulfilling community living requirements, such as moving around the house, eating, bathing, and maintaining continence (Kizir et al. 2024; Özbek Yazıcı and Kalaycı 2015). The primary desire of aging individuals is to perform these activities without assistance. However, age‐related changes in the musculoskeletal, cardiovascular, and neuromuscular systems complicate these functions, reducing daily living activities and leading to a loss of independence (Kankaya and Karadakovan 2017; Şahin et al. 2016). Degenerations, chronic disease complications, or unforeseen factors can result in partial or complete dependency, which diminishes autonomy, reduces quality of life, and increases the risk of abuse, neglect, morbidity, and mortality (United Nations 2024). Therefore, the ability to perform independent daily living activities is closely linked to preventing abuse and neglect (Kizir et al. 2024).

Enhancing elderly health necessitates addressing elder abuse and neglect from the perspectives of older adults and healthcare providers (United Nations 2024; WHO 2002). With Turkey's increasing elderly population, understanding the relationship between elder abuse, neglect, and daily living activity levels is crucial for enhancing intervention and care quality. Developing services and measures to protect older adults necessitates identifying the risks, circumstances, types, and mechanisms of abuse. Therefore, this study aims to make significant contributions to the field by using a comprehensive assessment tool to gather data on the types and perpetrators of elder abuse. Our study aims to provide substantial contributions to the field by using a comprehensive assessment tool to reveal data on the types of abuse and the perpetrators of elder abuse.

## 3 The Study

### 3.1 Aim

This study aimed to examine the relationship between daily living activity levels, sociodemographic characteristics, and experiences of elder abuse and neglect among elderly individuals residing in various provinces of Turkey. The study addressed the following hypotheses:

Hypothesis 1 *Elder abuse, particularly psychological abuse, is prevalent among individuals aged 65 and older*.

Hypothesis 2 *Dependency in daily living activities, as measured by Barthel Index scores, is associated with elder abuse and neglect*.

Hypothesis 3 *Sociodemographic characteristics such as gender, marital status, education level, and provinces influence the frequency of elder abuse and neglect*.

Hypothesis 4 *Family dynamics, including intrafamily conflict and low‐income family relationships, are associated with higher rates of elder abuse*.

## 4 Methods

### 4.1 Design

This descriptive, cross‐sectional study was conducted between January and August 2023 in seven provinces of Turkey: Tekirdağ, İzmir, Mersin, Bursa, Afyon, Kütahya, and Sinop.

### 4.2 Study Setting and Sampling

The inclusion criteria for the study were as follows: participants aged 65 years or older, those who voluntarily agreed to participate, individuals who could speak and understand Turkish, those oriented to place, time, and person, individuals without hearing or speech impairments, and those with a Mini‐Mental State Examination (MMSE) score between 24 and 30, indicating normal cognitive function. The exclusion criteria included neurological conditions (e.g., dementia or Alzheimer's disease) and psychiatric disorders (e.g., schizophrenia) that could affect mental status. Exclusion criteria included MMSE scores below 24, neurological conditions (e.g., dementia or Alzheimer's disease), and psychiatric disorders (e.g., schizophrenia) that could affect cognitive status. Cognitive eligibility was assessed through the MMSE and self‐reported information. When necessary, this was verified with input from family members living with the participants. Data collection was conducted in an environment where only the elderly participant was present, ensuring a distraction‐free and comfortable setting without the involvement of family members. The sample size was calculated using G Power 3.1.9.6 based on the estimated number of variables. With a 95% confidence level (*p* < 0.05), a small effect size (*d* = 0.2), and a critical *t*‐value of 1.967, it was planned to include 452 participants. To address the possible loss of 20% of the sample, researchers set the target sample size at 565 elderly individuals. The study ultimately included 448 participants, resulting in a participation rate of 79.76%.

### 4.3 Data Collection Tools

MMSE, developed by Folstein et al. (1975), is a widely used tool for assessing cognitive impairment (Folstein et al. 1975). Its Turkish version, validated by Güngen et al. (2002), evaluates orientation, memory, attention, calculation, and language, with a maximum score of 30. Scores are classified as follows: 24–30 (normal cognitive function), 18–23 (mild impairment), and 0–17 (severe impairment), with a sensitivity of 0.91 and specificity of 0.95 at the 23/24 threshold (Güngen et al. 2002). This study collected data from elderly participants with MMSE scores of 24 or higher to ensure the inclusion of individuals without significant cognitive impairment. Trained researchers conducted assessments following standardised procedures, and the MMSE was the primary tool used for mental evaluation.

Researchers prepared the Sociodemographic Data Collection Form based on relevant literature (Daşbaş et al. 2019; Pillemer et al. 2016; Çevik et al. 2021; Özmete 2016; Pak 2020; Storey 2020). The form comprised 23 questions covering age, gender, education level, number of children, place of residence, cohabitation status, income level, presence of chronic diseases, and other factors.

The Geriatric Mistreatment Scale, developed by Giraldo‐Rodríguez and Rosas‐Carrasco (2013), assesses various forms of mistreatment in elderly individuals (Giraldo‐Rodríguez and Rosas‐Carrasco 2013). Daşbaş et al. (2019) established the reliability and validity of its Turkish version. This scale evaluates various types and frequencies of elder abuse through 22 items categorised into five types: physical abuse (five items), psychological abuse (six items), neglect (four items), economic abuse (five items), and sexual abuse (two items). It also identifies the perpetrator (e.g., spouse, son, daughter), the onset of abuse, and the perpetrator's gender. Each item is scored in a binary format (0 = No = Abuse did not occur, 1 = Yes = Abuse occurred) to objectively assess mistreatment within the past year. For example, economic abuse is evaluated with the question, “Has someone used or is someone using your money without your consent?” Including a specific timeframe and explicit perpetrator identification ensures consistent categorization, reducing subjective interpretations and cultural biases. A positive response to any item indicates mistreatment, and the scale can be scored as a total abuse score or separately by category. Its original version demonstrated strong reliability (Cronbach's *α* = 0.80) with item‐total score correlations ranging from 0.27 to 0.58 (Daşbaş et al. 2019). In this study, the Cronbach's *α* value was 0.84, indicating high internal consistency. Permission to use the scale was obtained from Daşbaş via email.

The Barthel Activities of Daily Living Index, developed by Mahoney and Barthel (1965), assesses independence in daily life activities (Mahoney and Barthel 1965). The Turkish version's validity and reliability were established by Küçükdeveci et al. (2000). The index comprises 10 items scored from 0 to 100, indicating varying degrees of independence. These include feeding, bathing, grooming, dressing, bowel and bladder care, toilet use, transfers (bed to chair and back), mobility, and stairs. Scores range from 0 to 100, where 0–20 indicates full dependence, 21–61 severe dependence, 62–90 moderate dependence, 91–99 partial dependence, and 100 full independence (Küçükdeveci et al. 2000). The index has a Cronbach's *α* value of 0.93, indicating high internal consistency.

### 4.4 Data Collection

Data were collected from individuals aged 65 and older residing in Turkish provinces: Tekirdağ, İzmir, Mersin, Bursa, Afyon, Kütahya, and Sinop. Participants lived either in their own homes or with relatives. The study was conducted in seven provinces randomly selected by a lottery method from those with an elderly population rate of 8% or higher. Two trained researchers systematically collected data from households through visits in randomly chosen neighbourhoods. Researchers obtained verbal and written informed consent after explaining the study purpose and then administered the forms through face‐to‐face interviews. If the participants were illiterate, informed consent was obtained from the participants' legal guardians. The interviews were conducted comfortably and in a conducive setting in the older individual's residence. Older individuals who provided reliable information were selected for interviews; those with advanced dementia or Alzheimer's were excluded. Each form took approximately 15 min to complete per individual.

### 4.5 Data Analysis

Data analysis was performed using both SPSS 23.0 (Statistical Package for the Social Sciences) and the lme4 package in R. Descriptive statistical methods (mean, standard deviation, median, minimum–maximum, frequency) were used to evaluate the study data in SPSS. The chi‐squared test was applied to compare categorical variables, while the Shapiro–Wilk test was used to assess the normality of data distribution. For variables that did not exhibit normal distribution, the Spearman correlation test was used to analyse relationships, and the Mann–Whitney *U* test was used for comparing non‐normally distributed and non‐repeated data.

Further analysis used the lme4 package in R to explore the relationship between elder abuse and factors such as education level and health status. A multilevel logistic regression model was developed, with abuse as the dependent variable. Provinces and their sub‐locations (village, district, province) were included as random effects, while education level, health status, employment status, family relationships, and conflict were treated as fixed effects. Variance components (*τ*1, *τ*2), the intraclass correlation coefficient (ICC), Marginal *R* ^2^ (fixed effects), and Conditional *R* ^2^ (fixed and random effects combined) were calculated. Results were expressed as odds ratios (OR) with 95% confidence intervals (CI). A significance level of *p* < 0.05 was considered statistically significant.

### 4.6 Ethical Considerations

Ethical approval was obtained from the Non‐Interventional Ethics Committee of Izmir Democracy University (Decision No: 2023/01/‐07 dated January 04, 2023). The principles of the Declaration of Helsinki guided the study. The participants were informed of the study's goal and assured that their nonparticipation or withdrawal would not negatively affect them. Written consent was obtained from all participants. If the participants were illiterate, informed consent was obtained from their legal guardians.

## 5 Results

### 5.1 Demographic Characteristics of Older Individuals

The participants had a mean age of 71.20 ± 6.33 years (65–98), with 78.8% aged 65–74 and 51.6% identifying as male. A significant proportion of the participants were married (69.4%), and approximately half (40.8%) were literate. Geographically, 46.2% of the older adults lived in rural areas, while 32.1% resided in urban areas. Almost all participants (94.2%) reported having children; 30.4% had two children and 26.4% had three. Regarding employment status, 90.0% of the older adults were not actively working, while 10% were still employed. Financially, 50.2% did not have a stable income source. Half of those with an income reported that it covered their expenses, while 45% stated their income was insufficient. A large majority (80%) had social security coverage. Living arrangements varied, with 63.4% residing in their own homes, 21.2% in their spouse's residence, and 67.2% living with their spouse. Additionally, 17.6% lived alone, and 6.9% lived with their son. Family dynamics were generally positive, as 71% of the participants reported good relationships within their families, although 4.9% indicated poor relationships, and 8% reported intrafamily conflict (Table 1).

**TABLE 1:** Characteristics of the older adults and their scores on MMSE and Barthel Index (*n* = 448).

Variables	$\bar{X}\pm \mathrm{SD}$	Min–max
Age	71.20 ± 6.33	65–98
MMSE	27.11 ± 2.25	24–30
Barthel activities of daily living index	89.51 ± 19.03	10–100

	*n*	%
Gender		
Woman	217	48.4
Male	231	51.6
Age groups		
65–74 years old	353	78.8
75–84 years old	67	15.0
85 years and older	28	6.2
Marital status		
Married	311	69.4
Single	26	5.8
Widowed	111	24.8
Graduated school		
Illiterate	45	10.0
Literate	183	40.8
Elementary school	131	29.2
Middle school	51	11.4
High school	26	5.8
University	12	2.8
Place of residence		
Village	97	21.7
District	207	46.2
Provience	144	32.1
Name of province		
İzmir	105	23.4
Kütahya	48	10.7
Mersin	48	10.7
Sinop	28	6.3
Afyon	46	10.3
Bursa	48	10.7
Tekirdağ	125	27.9
Children		
Yes	422	94.2
No	26	5.8
Health insurance		
Uninsured	90	20.1
Social security institution	134	29.9
Bağ‐Kur	109	24.3
Pension fund	80	17.9
Green card	35	7.8
Presence of chronic disease		
Yes	321	71.7
No	127	28.3
Employment status		
Yes	45	10.0
No	403	90.0
Source of income		
Have	225	50.2
Not have	223	49.8
Monthly income		
Income < expenses	205	45.8
Income = expenses	223	49.8
Income > expenses	20	4.4
Living arrangements		
Own house	284	63.4
Spouse's house	95	21.2
Children's house	32	7.1
Rented house	24	5.4
Other	13	2.9
Co‐resident with the olderly		
Alone	79	17.6
Spose	301	67.2
Daughter	25	5.6
Son	31	6.9
Daughter‐in‐law	1	0.2
Son‐in‐law	1	0.2
Relatives	1	0.2
Caregiver	1	0.2
Other	8	1.8
Family relationships		
Very bad	10	2.2
Bad	22	4.9
Good	318	71.0
Very good	98	21.9
Intrafamily conflict		
Yes	36	8.0
No	412	92.0
Caregiver		
None	81	18.1
Family member	346	77.2
Neighbour	10	2.2
Paid caregiver	11	2.5
Self‐assessment of health		
Very good	188	42.0
Good	216	48.2
Fair	28	6.3
Bad	10	2.2
Very bad	6	1.3
Frequency of contact with family members		
I meet every day	197	44.0
I meet once a week	133	29.7
I meet once a month	75	16.7
I meet once every 6 months	26	5.8
I meet once a year	4	0.9
I never meet	13	2.9
Barthel activities of daily living index level		
Fully dependent	9	2.0
Severely dependent	32	7.1
Moderately dependent	96	21.4
Partially‐independent	30	6.7
Fully independent	281	62.7

*Note:* $\bar{X}\pm \mathrm{SD}$, total score mean ± standard deviation.

### 5.2 Health Status and Cognitive Function

A significant proportion (71.7%) of the participants reported chronic illnesses and 70.3% required regular medication. Notably, 77.2% received caregiving assistance from family members. The mean score on the MMSE for older participants in the study was 27.11 ± 2.25 (Min: 24, Max: 30). The mean score on the Barthel Index was 89.51 ± 19.03 (Min: 10, Max: 100). According to the distribution of scores on the Barthel Index, 2.0% were fully dependent, 7.1% were severely dependent, 21.4% were moderately dependent, 62.7% were fully independent, and 6.7% were partially independent. Correlation analysis showed a very weak positive correlation between MMSE and Barthel Index scores (*p* < 0.05, *r* = 0.105) and a weak negative correlation between age and Barthel Index scores (*p* < 0.001, *r* = −0.335) (Table 1).

### 5.3 Elder Abuse Prevalence and Characteristics

Of the participants, 22.8% (*n* = 102) reported experiencing at least one form of non‐sexual abuse. Specifically, 44.7% (*n* = 200) reported psychological abuse, 7.8% (*n* = 35) reported physical abuse, 5.3% (*n* = 24) reported economic abuse, and 1.12% (*n* = 5) reported experiencing neglect. The perpetrators of abuse across all types were frequently identified as sons, spouses, children, and other caregivers, predominantly male. Psychological abuse was most commonly reported as occurring ‘sometimes’ (29.1%) or ‘once’ (7.4%). Physical and economic abuse are generally reported as ‘once.’ The duration of psychological abuse is typically stated as 1 year or less, with some individuals (22.8%) unable to recall the duration (Table 2).

**TABLE 2:** Distribution of answers given to the sub‐dimensions of the Geriatric Mistreatment Scale^a^ (*n* = 448).

	Physical	Psychological	Economic	Sexual	Neglect
	*n* (%)	*n* (%)	*n* (%)	*n* (%)	*n* (%)
	35 (7.8)	200 (44.7)	24 (5.3)	0 (0.0)	5 (1.1)
Frequency of abuse and neglect					
Once	14 (3.0)	33 (7.4)	10 (2.2)	—	—
Sometimes	12 (2.7)	130 (29.1)	8 (1.7)	—	3 (0.7)
Always	—	13 (2.8)	1 (0.2)	—	—
No response	9 (2.0)	24 (5.3)	5 (1.0)	—	2 (0.4)
Duration of exposure to abuse and neglect					
1 year or less	4 (0.9)	97 (21.7)	5 (1.1)	—	2 (0.4)
Does not remember	10 (2.2)	103 (22.8)	2 (0.4)	—	2 (0.4)
Identity of the perpetrator					
Spouse	6 (1.3)	31 (7.1)	3 (0.7)	—	2 (0.4)
Daughter	—	14 (3.0)	3 (0.7)	—	—
Son	7 (1.5)	20 (4.4)	6 (1.3)	—	—
Spouse and children	1 (0.2)	44 (9.8)	2 (0.4)	—	—
Daughter‐in‐law	—	8 (1.5)	—	—	—
Son‐in‐law	—	1 (0.2)	—	—	—
Other relatives	3 (0.6)	14 (2.9)	4 (0.9)	—	—
Friend	3 (0.6)	9 (2.0)	—	—	1 (0.2)
Other (caregiver)	11 (2.1)	52 (11.6)	4 (0.9)	—	2 (0.4)
No response	—	—	—	—	—
Gender of the perpetrator					
Male	30 (6.6)	126 (28.1)	17 (3.6)	—	3 (0.7)
Woman	1 (0.2)	68 (15.1)	5 (1.1)	—	2 (0.4)
No response	—	—	—	—	—

	*n*	%
Suffered at least one type of abuse		
Yes	102	22.8
No	346	77.2
Total	448	100

^a^ More than one type of abuse may have been suffered by any single older individual.

### 5.4 Sociodemographic Factors and Abuse Correlations

The study results indicate statistically significant relationships between certain sociodemographic variables and elder abuse. Participants with a middle school education reported higher rates of physical (9.8%, *p* = 0.008), psychological (19.6%, *p* = 0.006), economic (7.8%, *p* = 0.011), and overall abuse (47.1%, *p* = 0.001) compared to other educational levels. Psychological abuse was significantly lower among single individuals compared to other groups (*p* = 0.030). Significant regional variations were observed in elder abuse rates across Turkey. Bursa reported the highest overall rate at 79.2%, including 47.9% for psychological abuse and 18.8% for physical abuse. Kütahya (25.0%) and Tekirdağ (24.8%) followed with similarly elevated rates. In contrast, lower rates were observed in İzmir, Mersin, Afyon, and Sinop, with Sinop recording the lowest overall rate at 7.1%. Economic abuse was more prevalent in İzmir, Kütahya, and Mersin but was rare in other provinces. Neglect cases were generally low, with Tekirdağ recording the highest rate at 24.8% (*p* < 0.001). Those with a positive perception of health reported significantly lower psychological abuse rates (*p* = 0.001), while employed individuals experienced higher rates of physical (*p* = 0.008), psychological (*p* = 0.011), and overall abuse (*p* = 0.002). The intrafamily conflict was associated with increased rates of physical abuse (*p* = 0.001) and overall abuse (*p* = 0.001), while low‐income family relationships were significantly linked to higher abuse rates (*p* = 0.001). In contrast, no significant relationships were found between elder abuse and certain sociodemographic variables. Gender, age groups, parental status, chronic disease presence, monthly income levels, and MMSE scores showed no significant associations with physical, psychological, or economic abuse or neglect (*p* > 0.05) (Table 3).

**TABLE 3:** Relationship between different types of abuse and descriptive variables (*N* = 448).

Variables	Physical abuse			Psychological abuse			Neglect			Economic abuse			Any type of abuse		
	No	Yes	*χ* ^2^/*p*	No	Yes	*χ* ^2^/*p*	No	Yes	*χ* ^2^/*p*	No	Yes	*χ* ^2^/*p*	No	Yes	*χ* ^2^/*p*
	*n* (%)	*n* (%)		*n* (%)	*n* (%)		*n* (%)	*n* (%)		*n* (%)	*n* (%)		*n* (%)	*n* (%)	
Gender															
Female	207 (95.4)	10 (4.6)	*χ* ^2^ = 3.059 *p* = 0.080	197 (90.8)	20 (9.2)	*χ* ^2^ = 2.112 *p* = 0.146	212 (98.1)	4 (1.9)	*χ* ^2^ = 2.481 *p* = 0.054	213 (98.2)	4 (1.8)	*χ* ^2^ = 0.216 *p* = 0.717	165 (76.0)	52 (24.0)	*χ* ^2^ = 0.342 *p* = 0.575
Man	227 (98.3)	4 (1.7)		218 (94.4)	13 (5.6)		231 (100.0)	0 (0.0)		228 (98.7)	3 (1.3)		181 (78.4)	50 (21.6)	
Age groups															
65–74 years old	324 (78.8)	11 (3.1)	*χ* ^2^ = 0.352 *p* = 0.883	327 (92.6)	26 (7.4)	*χ* ^2^ = 0.853 *p* = 0.656	398 (98.9)	4 (1.1)	*χ* ^2^ = 0.395 *p* = 1.000	346 (98.0)	7 (2.0)	*χ* ^2^ = 0.737 *p* = 0.748	271 (76.8)	82 (23.2)	*χ* ^2^ = 1.478 *p* = 0.478
75–84 years old	65 (97)	2 (3.0)		63 (94.0)	4 (6.0)		67 (100.0)	0 (0.0)		67 (100.0)	0 (0.0)		55 (82.1)	12 (17.9)	
85 years and older	27 (96.4)	1 (3.6)		25 (89.3)	3 (10.7)		28 (100.0)	0 (0.0)		28 (100.0)	0 (0.0)		20 (71.4)	8 (28.6)	
Marital status															
Married	302 (97.1)	9 (2.9)	*χ* ^2^ = 0.652 *p* = 0.707	292 (93.9)	19 (6.1)	*χ* ^2^ = 6.398 *p* = 0.030*	309 (99.4)	2 (0.6)	*χ* ^2^ = 1.653 *p* = 0.434	308 (99.0)	3 (1.0)	*χ* ^2^ = 3.347 *p* = 0.165	242 (77.8)	69 (22.2)	*χ* ^2^ = 0.214 *p* = 0.898
Single	25 (96.2)	1 (3.8)		26 (100.0)	0 (0.0)		26 (100.0)	0 (0.0)		26 (100.0)	0 (0.0)		20 (76.9)	6 (23.1)	
Widowed	107 (96.4)	4 (3.6)		97 (87.4)	14 (12.6)		108 (98.2)	2 (1.8)		107 (96.4)	4 (3.6)		84 (75.7)	27 (24.3)	
Graduated school															
Illiterate	43 (95.6)	2 (4.4)	*χ* ^2^ = 13.432 *p* = 0.008*	41 (91.1)	4 (8.9)	*χ* ^2^ = 15.101 *p* = 0.006*	45 (100.0)	0 (0.0)	*χ* ^2^ = 5.931 *p* = 0.228	44 (97.8)	1 (2.2)	*χ* ^2^ = 11.975 *p* = 0.011*	33 (73.3)	12 (26.7)	*χ* ^2^ = 19.571 *p* = 0.001**
Literate	182 (99.5)	1 (0.5)		177 (6)	6 (3.3)		182 (100.0)	0 (0.0)		183 (100.0)	0 (0.0)		147 (80.3)	36 (19.7)	
Elementary school	126 (96.2)	5 (3.8)		119 (90.8)	12 (9.2)		128 (97.7)	3 (2.3)		129 (98.5)	2 (1.5)		105 (80.2)	26 (19.8)	
Middle school	46 (90.2)	5 (9.8)		41 (80.4)	10 (19.6)		50 (98.0)	1 (2.0)		47 (92.2)	4 (7.8)		27 (52.9)	24 (47.1)	
High school	26 (100.0)	0 (0.0)		25 (96.2)	1 (3.8)		26 (100.0)	0 (0.0)		26.0 (100.0)	0 (0.0)		23 (88.5)	3 (11.5)	
University	11 (91.7)	1 (8.3)		12 (100.0)	0 (0.0)		12 (100.0)	0 (0.0)		12 (100.0)	0 (0.0)		11 (91.7)	1 (8.3)	
Children															
Yes	410 (97.2)	12 (2.8)	*χ* ^2^ = 0.637 *p* = 0.192	392 (92.9)	30 (7.1)	*χ* ^2^ = 0.704 *p* = 0.427	417 (99.0)	4 (1.0)	*χ* ^2^ = 0.249 *p* = 1.000	415 (98.3)	7 (1.7)	*χ* ^2^ = 0.704 *p* = 0.427	323 (76.5)	99 (23.5)	*χ* ^2^ = 1.979 *p* = 0.228
No	24 (92.3)	2 (7.7)		23 (87.5)	3 (11.5)		26 (100.0)	0 (0.0)		26 (100.0)	0 (0.0)		23 (88.5)	3 (11.5)	
Place of residence															
Village	92 (94.8)	5 (5.2)	*χ* ^2^ = 2.844 *p* = 0.219	91 (93.8)	6 (6.2)	*χ* ^2^ = 1.718 *p* = 0.436	97 (100.0)	0 (0.0)	*χ* ^2^ = 1.135 *p* = 0.688	96 (99.0)	1 (1.0)	*χ* ^2^ = 1.480 *p* = 0.546	79 (81.4)	18 (18.6)	*χ* ^2^ = 17.280 *p* = 0.001**
District	200 (96.6)	7 (3.4)		188 (90.8)	19 (9.2)		204 (98.6)	3 (1.4)		202 (97.6)	5 (2.4)		142 (68.6)	65 (31.4)	
Province	142 (98.6)	2 (1.4)		136 (94.4)	8 (5.6)		142 (99.3)	1 (0.7)		143 (99.3)	1 (0.7)		125 (86.8)	19 (13.2)	
Name of provinces															
İzmir	104 (99.0)	1 (1.09)	*χ* ^2^ = 45.764 *p* = 0.001**	102 (97.1)	3 (2.9)	*χ* ^2^ = 131.957 *p* = 0.001**	104 (100.0)	0 (0.0)	*χ* ^2^ = 17.948 *p* = 0.009*	104 (99.0)	1 (1.0)	*χ* ^2^ = 17.948 *p* = 0.013	94 (89.5)	11 (10.5)	*χ* ^2^ = 111.035 *p* = 0.001**
Kütahya	46 (95.8)	2 (4.2)		45 (93.8)	3 (6.2)		47 (97.9)	1 (2.1)		47 (97.9)	1 (2.1)		36 (75.0)	12 (25.0)	
Mersin	47 (97.6)	1 (2.1)		46 (95.8)	2 (4.2)		48 (100.0)	0 (0.0)		48 (100.0)	0 (0.0)		44 (91.7)	4 (3.9)	
Sinop	28 (100.0)	0 (0.0)		27 (96.4)	1 (3.6)		28 (100.0)	0 (0.0)		28 (100.0)	0 (0.0)		26 (92.9)	2 (7.1)	
Afyon	45 (97.8)	1 (2.2)		45 (97.8)	1 (3.6)		468 (100.0)	0 (0.0)		45 (97.8)	1 (2.2)		42 (91.3)	4 (8.7)	
Bursa	39 (81.3)	9 (18.8)		25 (52.1)	23 (47.9)		45 (93.7)	3 (6.3)		44 (91.7)	4 (8.3)		10 (20.8)	38 (79.2)	
Tekirdağ	125 (100.0)	0 (0.0)		125 (100.0)	0 (0.0)		125 (100.0)	0 (0.0)		125 (100.09)	0 (0.0)		94 (75.2)	31 (24.8)	
Perception of health															
Very good	187 (99.5)	1 (0.5)	*χ* ^2^ = 11.311 *p* = 0.018*	185 (98.4)	3 (1.6)	*χ* ^2^ = 20.737 *p* = 0.001**	188 (100.0)	0 (0.0)	*χ* ^2^ = 6.779 *p* = 0.202	187 (95.5)	1 (0.5)	*χ* ^2^ = 4.514 *p* = 0.358	168 (89.4)	20 (10.6)	*χ* ^2^ = 30.015 *p* = 0.001**
Good	206 (95.4)	10 (4.6)		191 (88.4)	25 (11.6)		212 (98.6)	3 (1.4)		211 (97.7)	5 (2.3)		148 (68.5)	68 (31.5)	
Moderate	25 (95.4)	3 (10.7)		24 (85.7)	4 (14.3)		27 (96.4)	1 (3.6)		27 (96.4)	1 (3.6)		20 (71.4)	8 (28.6)	
Bad	10 (100.0)	0 (0.0)		10 (100.0)	0 (0.0)		10 (100.0)	0 (0.0)		10 (100.0)	0 (0.0)		6 (60.0)	4 (40.0)	
Very bad	6 (100.0)	0 (0.0)		5 (83.3)	1 (16.7)		6 (100.0)	0 (0.0)		6 (100.0)	0 (0.0)		4 (66.7)	2 (33.3)	
Presence of chronic disease															
Yes	311 (96.9)	10 (3.1)	*χ* ^2^ = 0.498 *p* = 1.000	300 (93.5)	21 (6.6)	*χ* ^2^ = 1.127 *p* = 0.288	317 (99.1)	3 (0.9)	*χ* ^2^ = 1.023 *p* = 1.000	314 (97.8)	7 (2.2)	*χ* ^2^ = 1.574 *p* = 0.199	245 (76.3)	76 (23.7)	*χ* ^2^ = 9.572 *p* = 0.532
No	123 (96.9)	4 (3.1)		115 (90.6)	12 (9.4)		126 (99.2)	1 (0.8)		127 (100.0)	0 (0.0)		101 (79.5)	26 (20.5)	
Employment															
No	394 (97.8)	9 (2.2)	*χ* ^2^ = 7.810 *p* = 0.008*	378 (93.8)	25 (6.2)	*χ* ^2^ = 6.342 *p* = 0.011	399 (99.3)	3 (0.7)	*χ* ^2^ = 0.026 *p* = 0.347	399 (99.0)	4 (1.0)	*χ* ^2^ = 5186 *p* = 0.025	320 (79.4)	83 (20.6)	*χ* ^2^ = 0.365 *p* = 0.002*
Yes	40 (88.9)	5 (11.1)		37 (82.2)	8 (17.8)		44 (97.8)	1 (2.2)		42 (93.3)	3 (6.7)		26 (57.8)	19 (42.2)	
Monthly income															
Income < expenses	201 (98.0)	4 (2.0)	*χ* ^2^ = 2.145 *p* = 0.311	197 (96.1)	8 (3.9)	*χ* ^2^ = 7.382 *p* = 0.022*	203 (99.5)	1 (0.5)	*χ* ^2^ = 1.125 *p* = 0.688	203 (99.0)	2 (1.0)	*χ* ^2^ = 1.073 *p* = 0.603	153 (74.6)	52 (25.4)	*χ* ^2^ = 1.650 *p* = 0.460
Income = expenses	213 (95.5)	10 (4.5)		199 (89.2)	24 (10.8)		220 (98.7)	3 (1.3)		218 (97.8)	5 (2.2)		176 (78.9)	47 (21.1)	
Income > expenses	20 (100.0)	0 (0.0)		19 (95.0)	1 (5.0)		20 (100.0)			20 (100.0)	0 (0.0)		17 (85.0)	3 (15.0)	
Family relationships															
Very bad	10 (100.0)	0 (0.0)	*χ* ^2^ = 0.249 *p* = 1.000	10 (100.0)	0 (0.0)		10 (100.0)	0 (0.0)	*χ* ^2^ = 4.291 *p* = 0.289	10 (100.0)	0 (0.0)	*χ* ^2^ = 9.821 *p* = 0.071	7 (70.0)	3 (30.0)	*χ* ^2^ = 17.070 *p* = 0.001**
Bad	18 (81.8)	4 (18.2)		16 (72.7)	6 (27.3)	*χ* ^2^ = 10.011	21 (95.5)	1 (4.5)		20 (90.9)	2 (9.1)		11 (50.0)	11 (50.0)	
Good	308 (96.9)	10 (3.1)		297 (93.4)	21 (6.6)	*p* = 0.018	314 (99.1)	3 (0.9)		313 (98.4)	5 (1.6)		241 (75.8)	77 (24.2)	
Very good	98 (100.0)	0 (0.0)		92 (93.9)	6 (6.1)		98 (100.0)	0 (0.0)		98 (100.0)	0 (0.0)		87 (88.8)	11 (11.2)	
Intrafamily conflict															
No	404 (98.1)	8 (1.9)	*χ* ^2^ = 19.097 *p* = 0.001**	384 (93.2)	28 (6.8)	*χ* ^2^ = 1.512	408 (99.3)	3 (0.7)	*χ* ^2^ = 1.565 *p* = 0.286	407 (98.8)	5 (1.2)	*χ* ^2^ = 1.726 *p* = 0.102	328 (79.6)	84 (20.4)	*χ* ^2^ = 16.509 *p* = 0.001**
Yes	30 (6.9)	6 (16.7)		31 (86.1)	5 (13.9)	*p* = 0.170	35 (97.2)	1 (2.8)		34 (94.4)	2 (5.6)		18 (50.0)	18 (50.0)	
Frequency of contact with family members															
Every day	189 (95.9)	8 (4.1)	*χ* ^2^ = 21.412 *p* = 0.010*	175 (88.8)	22 (11.2)		194 (99.0)	2 (1.0)	*χ* ^2^ = 8.442 *p* = 0.166	194 (99.0)	2 (2.0)	*χ* ^2^ = 12.566 *p* = 0.014*	151 (76.6)	46 (23.4)	*χ* ^2^ = 6.364 *p* = 0.250
Once a week	131 (95.8)	2 (1.5)		128 (96.2)	5 (3.8)		133 (100.0)	0 (0.0)		133 (100.0)	0 (0.0)		108 (81.2)	25 (18.8)	
Once a month	75 (100.0)	0 (0.0)		73 (97.3)	2 (2.7)		74 (98.7)	1 (1.03)		74 (98.7)	1 (1.3)		55 (73.3)	20 (26.7)	
Once every 6 months	25 (96.2)	1 (83.8)		24 (92.3)	2 (7.7)	*χ* ^2^ = 10.682	26 (100.0)	0 (0.0)		26 (100.0)	0 (0.0)		22 (84.6)	4 (15.4)	
Once a year	4 (100.0)	0 (0.0)		4 (100.0)	0 (0.0)	*p* = 0.041*	4 (100.0)	0 (0.0)		4 (100.0)	0 (0.0)		2 (50.0)	2 (50.0)	
Never meet	10 (76.9)	3 (23.1)		11 (84.6)	2 (15.4)		12 (92.3)	1 (7.7)		12 (92.3)	1 (7.7)		8 (61.5)	5 (38.5)	

	No	Yes	*Z*/*t*	No	Yes	*Z*/*t*	No	Yes	*Z*/*t*	No	Yes	*Z*/*t*	No	Yes	*Z*/*p*
	$\bar{X}\pm \mathrm{SD}$	$\bar{X}\pm \mathrm{SD}$		$\bar{X}\pm \mathrm{SD}$	$\bar{X}\pm \mathrm{SD}$		$\bar{X}\pm \mathrm{SD}$	$\bar{X}\pm \mathrm{SD}$		$\bar{X}\pm \mathrm{SD}$	$\bar{X}\pm \mathrm{SD}$		$\bar{X}\pm \mathrm{SD}$	$\bar{X}\pm \mathrm{SD}$	
Age	71.2 ± 6.3	71.64 ± 5.74	*Z* = −0.614 *p* = 0.539	71.0 ± 6.3	72.7 ± 6.2	*Z* = −2.009 *p = 0*.045*	71.2 ± 6.3	67.5 ± 0.5	*Z* = −1.187 *p* = 0.235	71.2 ± 6.3	69.8 ± 2.1	*Z* = −0.318 *p* = 0.751	71.1 ± 6.3	71.3 ± 6.4	*Z* = −0.372 *p* = 0.710
MMSE	27.3 ± 2.1	27.1 ± 2.2	*Z* = −0.297 *p* = 0.766	27.1 ± 2.2	26.9 ± 2.3	*Z* = −0.552 *p* = 0.581	27.1 ± 2.2	27.7 ± 2.6	*Z* = −0.498 *p* = 0.619	27.1 ± 2.2	27.1 ± 2.1	*Z* = −0.024 *p* = 0.981	27.1 ± 2.2	27.0 ± 2.3	*Z* = −0.450 *p* = 0.653
Bartel index	89.5 ± 18.9	88.9 + 21.5	*Z* = −0.259 *p* = 0.796	89.5 ± 18.7	89.0 ± 18.7	*Z* = −0.320 *p* = 0.749	89.3 ± 19.1	100.0 ± 0.0	*Z* = −1.496 *p* = 0.135	87.7 ± 19.0	85.7 ± 19.8	*Z* = −0.534 *p = 0*.593	89.1 ± 19.1	90.7 ± 18.5	*Z* = −1.386 *p* = 0.166

*Note:* $\bar{X}\pm \mathrm{SD}$, total score mean ± standard deviation; *Z*, Mann–Whitney test.

Abbreviation: MMSE, Mini Mental State Examination.

* *p* < 0.05 level (2‐tailed).

** *p* < 0.001 level (2‐tailed).

Correlation analysis examined the relationships between MMSE scores, age, abuse exposure, and Barthel Index scores. The strength of these relationships was evaluated using the guidelines provided by Schober and Schwarte (2018), which classify correlations as follows: very weak (0.00–0.19), weak (0.20–0.39), moderate (0.40–0.69), strong (0.70–0.89), and very strong (0.90–1.00) (Schober and Schwarte 2018). No significant relationship was found between dependency in daily living activities and experiences of elder abuse (*p* > 0.05). A very weak positive correlation was identified between MMSE and Barthel Index scores (*p* < 0.05, *r* = 0.105), and a weak negative correlation was found between age and Barthel Index scores (*p* < 0.001, *r* = −0.335) (not shown in the data table).

### 5.5 Factors Affecting Abuse Risk and Multilevel Analysis Results

The multilevel analysis revealed significant factors affecting abuse risk. Individuals experiencing intrafamily conflict had a 3.93 times higher risk of abuse (*p* = 0.018). Self‐assessment of health also emerged as a key determinant, with those rating their health as “good” having a 3.56 times higher risk and those rating it as “bad” or “very bad” exhibiting a 12.97 times higher risk (*p* < 0.001). Interestingly, individuals rating their family relationships as “good” also showed an increased risk of abuse (OR = 3.35, *p* = 0.009), while no significant association was found for those with “bad” or “very bad” relationships (*p* = 0.214). Employment status and education level did not significantly affect abuse risk (*p* > 0.05). Random effects analysis showed substantial variability in abuse risk across provinces and their sub‐locations (*τ*1 = 1.56; *τ*2 = 0.71), highlighting the role of spatial differences. The intraclass correlation coefficient (ICC = 0.41) indicated a moderate impact of random effects. Fixed effects explained 15.4% of the variance in abuse risk (Marginal *R* ^2^ = 0.154), while including random effects increased this to 50% (Conditional *R* ^2^ = 0.500). These findings underscore the importance of considering individual factors, such as health perception, intrafamily conflict, and family dynamics, alongside spatial differences in evaluating abuse risk (Table 4).

**TABLE 4:** Multivariate logistic regression of health and social determinants.

Independent variables	Odds ratios	CI	*p*
Intercept	0.03	0.01–0.20	0.001**
Education level [primary school] (Ref = high school + university graduate)	1.20	0.30–5.71	0.786
Education level [literate] (Ref = high school + university graduate)	0.96	0.25–3.70	0.947
Education level [illiterate] (Ref = high school + university graduate)	1.32	0.30–5.71	0.712
Education level [middle school graduate] (Ref = high school + university graduate)	1.05	0.25–4.53	0.943
Self‐assessment of health [good] (Ref = very good)	3.56	1.81–7.02	0.001**
Self‐assessment of health [bad + very bad] (Ref = verygood)	12.97	3.22–52.34	0.001**
Self‐assessment of health [fair] (Ref = very good)	4.16	1.24–13.98	0.021*
Employment status [employed] (Ref = unemployed)	1.27	0.44–3.62	0.661
Family relationships [good] (Ref = very good)	3.35	1.34–8.36	0.009*
Family relationships [bad + very bad] (Ref = very good)	2.44	0.60–9.96	0.214
Intrafamily conflict [present] (Ref = absent)	3.93	1.26–12.29	0.018*

Abbreviations: CI, confidence interval; OR, odds ratio; Ref, reference category.

* *p* < 0.05.

** *p* < 0.001.

## 6 Discussion

Elder abuse is a critical issue that demands attention from healthcare systems, social welfare organisations, policymakers, and the public (Acierno et al. 2017; Pillemer et al. 2016). Understanding its prevalence and associated risk factors is crucial; however, challenges in reporting persist. Only a small proportion of cases—around 5.0%—are reported, making it difficult to determine the true prevalence and risk factors (Yılmaz et al. 2022).

The mean age of participants in our study was 71.20 ± 6.33 years, with 78.8% aged 65–74 and 51.6% being male. Previous studies have reported similar trends. For instance, Dabas and Isikhan (2019) found a mean age of 75 years, with 46.6% of participants aged 65–74 and 68.6% male (Dabas and Isikhan 2019). Pak (2020) reported that 48.0% were aged 60–69, while 65.8% were male (Pak 2020). Similarly, Özer and Tanriverdi (2023) observed that 66.0% were aged 60–74, and 42.5% were male (Özer and Tanriverdi 2023). Çevik et al. (2021) reported half of their participants were aged 65–74, and half were male (Çevik et al. 2021). Yılmaz et al. (2022) and Kulakçı Altıntaş and Korkmaz Aslan (2020) noted mean ages of 71 and 70 years, respectively (Kulakçı Altıntaş and Korkmaz Aslan 2020; Yılmaz et al. 2022). These findings suggest a consistent demographic trend in older populations across studies.

In our study, 22.8% of older individuals reported experiencing at least one form of abuse. The most prevalent type was psychological abuse (44.7%), followed by physical abuse (7.8%) and economic abuse (5.3%). Most perpetrators were male relatives, primarily sons or spouses. These findings align with previous studies that report elder abuse prevalence rates between 13.3% and 28.5%. For example, Gursoy and Kara (2020) documented a prevalence of 28.5% in Çanakkale, while Yılmaz et al. (2022) reported lower rates in Kütahya, including 6.9% for psychological abuse, 6.6% for physical abuse, and 1.5% for economic abuse in Turkey (Gursoy and Kara 2020; Yılmaz et al. 2022). Similarly, another study identified psychological abuse as the most frequent type (18.2%) in southern Turkey (Yalçın Gürsoy and Uçan Yamaç 2024). Globally, higher abuse rates have been reported. For instance, Torres‐Castro et al. (2018) highlighted psychological and financial abuse as the most common forms worldwide, while Macassa et al. (2013) observed psychological abuse rates of 29.7% in Sweden, 27.1% in Germany, and 24.6% in Lithuania (Torres‐Castro et al. 2018; Macassa et al. 2013). Rosay and Mulford (2017) revealed that psychological abuse is the most common form of mistreatment among individuals aged 70 and older, affecting 12.1% of this group. Notably, 57.1% of these incidents were committed by non‐relatives (Rosay and Mulford 2017). Similarly, one study reported psychological abuse as the most frequent type, with a prevalence of 30.6%, in older women in Mexico City (Vilar‐Compte et al. 2018). Psychological abuse frequently involves repeated verbal aggression, derogatory remarks regarding age or health, and social isolation (Conrad et al. 2011). However, isolated incidents, such as a single argument, should not be considered abuse. The higher prevalence of psychological abuse observed in our study, compared to others, may be attributed to cultural and social dynamics that shape how abuse is perceived and reported. In some contexts, certain normative behaviours may be misinterpreted as abuse, leading to increased reporting rates. Furthermore, differences in measurement tools, participants' sociocultural backgrounds, and regional variations may also contribute to these discrepancies. Additionally, the specific wording and structure of the assessment tool used in our study might have facilitated the identification of psychological abuse more effectively than tools used in other studies. These findings emphasise the need for further research to understand how cultural differences influence perceptions of psychological abuse. Future studies should focus on qualitative approaches to explore these cultural variations in greater depth.

Older adults who rely on others for daily living activities face a greater risk of abuse and neglect (Pillemer et al. 2016). Difficulty or inability to perform these activities increases their dependence on family members and caregivers. This reliance limits their ability to seek help or defend themselves. Dependency often leads to social isolation, learned helplessness, and hesitation in reporting abuse, which collectively heightens the risk of mistreatment (Pillemer et al. 2021; Zhang et al. 2022). Our study's Barthel Index scores showed that 2.0% of participants were fully dependent, 7.1% were severely dependent, and 21.4% were moderately dependent. However, no significant link was found between dependency in daily living activities and experiences of elder abuse and neglect. Comparatively, Kankaya and Karadakovan (2017) reported 5.0% entirely dependent and 12.0% severely dependent older adults (Kankaya and Karadakovan 2017). Kizir et al. (2024), on the other hand, identified 43.8% as moderately dependent, 29.4% as partially independent, and 7.2% as severely dependent, observing a decrease in abuse with higher activity levels (Kizir et al. 2024). Similarly, Aslan and Erci (2020) noted a positive relationship between dependency and elder abuse, while Atim et al. (2023) linked moderate to full functional dependence with various types of abuse, except sexual abuse (Aslan and Erci 2020; Atim et al. 2023). However, studies by Choksomngam et al. (2023), Dong and Simon (2010), and Comijs et al. (1998) found no association between daily living activities and elder abuse or neglect (Choksomngam et al. 2023; Dong and Simon 2010; and Comijs et al. 1998). Our study found no significant association between dependency in daily living activities and elder abuse. This outcome can be attributed to the multifaceted nature of abuse, influenced by socioeconomic factors, family dynamics, caregiver stress, and cultural norms. Additionally, the predominance of independent participants in the sample might have limited the findings. In predominantly Muslim societies like Turkey, where strong family bonds are prevalent, dependent older adults often receive increased care and attention rather than being subjected to mistreatment. Furthermore, caregivers' ability to manage stress and access to professional care services are critical factors that may reduce the risk of abuse. Methodological limitations may have weakened the observed link between dependency and abuse. These limitations include individuals' reluctance to report abuse and the use of cross‐sectional study designs. Moreover, individual resilience factors, including social support and self‐esteem, can mitigate the risk of abuse even in cases of high dependency. Longitudinal studies are necessary to gain a more comprehensive understanding of the dynamics at play.

This study emphasised the significant role of socioeconomic, personal, and social factors in the occurrence of elder abuse. The findings revealed that elderly individuals with lower education levels, poor financial status, and health problems, particularly those residing in rural areas, were more vulnerable to abuse. Our study demonstrated that intrafamily conflict increased the risk of elder abuse by 3.93 times, while poor self‐perceived health raised this risk by 12.97 times. Interestingly, the increased risk observed in “good” family relationships may reflect underlying tensions or hidden vulnerabilities, highlighting the complexity of family dynamics. This phenomenon, rooted in the intricacies of family interactions and underlying issues that often remain concealed, may result in heightened expectations from elderly individuals, potentially causing caregiver stress and burnout. Therefore, conducting in‐depth qualitative research is recommended to better understand the nature of family relationships and uncover underlying factors contributing to elder abuse.

Elder abuse is widely recognised as a form of domestic violence, as it often occurs within family settings (WHO 2022). In Turkey, domestic violence has become a significant societal issue, primarily affecting women and rooted in gender inequality (Council of Europe 2017). Research shows that women who face intimate partner violence earlier in life remain vulnerable in old age, especially in households where patriarchal norms and financial dependence create power imbalances (Taşkesen 2017). The normalisation of domestic violence can lead to continued abuse of elderly women, increasing their risk of psychological, economic, and physical mistreatment (Greulich and Dasré 2022).

However, elder abuse affects both genders. Gendered perceptions, such as viewing men as superior and assigning caregiving roles to women, increase women's vulnerability to abuse and neglect. In Turkey, the vast majority of elderly women do not have retirement security and remain economically dependent on their children. Additionally, economic dependence and societal pressures further silence them, making it harder for them to seek help (Taşkesen 2017). Cultural norms, such as the expectation to “maintain the marriage,” patriarchal values, social pressure, and lack of self‐confidence, normalise violence and discourage victims from seeking assistance (Greulich and Dasré 2022; Sarıtaş 2023). Similarly, older men may also experience violence and neglect due to economic dependence, physical frailty, and social isolation. However, because domestic violence is primarily framed as a women's issue, the victimisation of elderly men is often overlooked. The societal expectation that men should be “strong” and “independent” discourages them from reporting psychological, economic, or physical abuse. Additionally, the lack of support for men restricts their ability to seek help (Sarıtaş 2023). Future research should examine gender‐specific barriers to reporting and the impact of social norms.

In our study, although women aged 65 and older reported higher rates of abuse, no statistically significant gender‐based differences were found. Many women remain emotionally attached to or financially dependent on their abusers, making it difficult for them to seek help (Dong 2015). Reliable data on elder abuse, mistreatment, and neglect largely depend on victims coming forward; however, many older individuals refrain from doing so due to shame, social pressure, a desire to maintain family ties, feelings of helplessness, or fear of further violence (Kulakçı Altıntaş and Korkmaz Aslan 2020; Pillemer et al. 2016; Storey 2020). Moreover, state policies, official discourse, and gender biases within the judicial system reinforce gender inequality, restricting women's fundamental rights and normalising violence. Similarly, older men often avoid reporting abuse due to stigma, fear of appearing weak, and lack of support services (Sahin 2022). While these challenges are often discussed in relation to women, older men also face significant barriers to reporting abuse. They frequently avoid disclosure due to stigma, fear of appearing weak, and lack of support services.

In addition to the socioeconomic and health‐related factors emphasised in our study, the broader literature identifies additional key risk factors for elder abuse, including poor physical and mental health, cognitive impairment, financial dependency, marital and parental status, medication use, social isolation, heightened emotional intensity, and inadequate support systems (Atim et al. 2023; Gursoy and Kara 2020; Mercier et al. 2020; Santos‐Rodrigues et al. 2022; Storey 2020; Yılmaz et al. 2022). These findings highlight the multifaceted nature of elder abuse and the necessity of a more comprehensive analysis to capture regional variations in abuse patterns. Our study underscores significant regional differences in elder abuse risk. For instance, Bursa exhibits the highest abuse rate at 79.2%, while Sinop, despite having a large elderly population, reports the lowest at 7.1%. These disparities may stem from differences in urbanisation, economic conditions, and family structures. In industrialised cities like Bursa, economic stress and the prevalence of nuclear families may weaken intergenerational support, increasing the risk of abuse. Conversely, in rural areas like Sinop, extended family structures may provide stronger social support, reducing abuse prevalence. Economic abuse appears more common in metropolitan areas such as İzmir, Kütahya, and Mersin, likely due to higher financial dependency and intergenerational economic ties, whereas neglect is more frequently reported in Tekirdağ, possibly due to limited caregiving resources and shifting family dynamics. These findings align with prior studies linking urbanisation, industrialisation, and nuclear family prevalence to regional disparities in elder abuse (Banerjee et al. 2020; Mercier et al. 2020).

In western Turkey, industrialisation and urbanisation are well‐developed, which, along with the prevalence of the nuclear family structure, can significantly impact the living arrangements and care processes of elderly individuals. When older adults move in with their children, it often leads to three generations living together. However, this situation may impose additional burdens in terms of space, time, and caregiving responsibilities (Taşkesen 2017). In Turkey's Southeastern and Eastern Anatolia regions, elderly care remains largely family‐based, resulting in fewer institutional care options. Strong privacy norms and communal ties can make detecting and reporting elder abuse more difficult, while economic hardships and inadequate social services further limit access to external support systems (Pillemer et al. 2016; Şahinli and Tarım 2019). Existing research primarily focuses on urban areas, leaving rural elderly populations understudied (Dabas and Isikhan 2019; Yalçın Gürsoy and Uçan Yamaç 2024; Yılmaz et al. 2022). Addressing these gaps requires region‐specific policies and field studies to develop targeted protection and support mechanisms for older adults.

Elder mistreatment is not just an immediate trauma but a serious social issue with lasting health and psychological effects. The NEMS Wave II study by Acierno et al. (2017) found a strong link between elder mistreatment and depression, post‐traumatic stress disorder, and poor health outcomes (Acierno et al. 2017). While NEMS I focused on the prevalence and risk factors of mistreatment (Laumann et al. 2008), NEMS II examined its long‐term impact, emphasising the crucial role of social support in reducing these negative effects (Acierno et al. 2017). Despite these findings, systematic research on the long‐term consequences of elder mistreatment in Turkey remains limited, making it difficult to develop effective interventions. Without longitudinal studies, fully understanding the scope of the issue and implementing sustainable solutions remains a challenge. Expanding research in this field is essential not only for developing strong preventive policies but also for ensuring a better quality of life for older adults.

### 6.1 Limitations and Strengths

Our study has some limitations. The first limitation is the reliance on self‐reported data, which may introduce bias. Participants may underreport experiences of abuse due to fear of retaliation, feelings of embarrassment, or cultural expectations. Additionally, victims may feel particularly reluctant to disclose abuse if someone close to them, who could potentially be a perpetrator, is required to consent on their behalf. While interviews were conducted in the older individual's residence at a convenient time and in a private setting that ensured confidentiality, it should be considered the potential for underreporting. The second limitation is the study's geographic limitation to specific provinces in Turkey, which may limit its generalisability. Thirdly, excluding individuals with severe cognitive impairments or psychiatric conditions may overlook a vulnerable group at high risk of abuse and neglect. Fourthly, the cross‐sectional design makes it difficult to establish causal relationships between sociodemographic factors and elder abuse. Despite these limitations, the study has notable strengths. It employs a comprehensive approach with validated tools to gather detailed data on cognitive function, daily living activities, and abuse prevalence. The study's multifaceted analysis includes various sociodemographic factors, providing a thorough understanding of elder abuse and neglect.

### 6.2 Recommendations for Further Research

The high prevalence of psychological abuse highlights the need for additional assessment tools to address its complex nature. Although the Geriatric Mistreatment Scale has unique features and strengths, it does not include the professional perspective on the older individual's responses, such as assessing behaviours that align with or contradict their statements. Therefore, future studies should incorporate questions that consider the participant's responses and the evaluations of professionals and/or researchers. Future studies should examine the prevalence and regional differences in elder abuse through large‐scale and nationally representative samples in Turkey. The impact of cognitive impairments on elder abuse risk should be investigated to understand how cognitive decline increases vulnerability. Longitudinal and qualitative research is essential to explore the underlying causes of elder mistreatment and its impact on individuals. Furthermore, studies should evaluate the effectiveness of elder care services and social support systems, as well as investigate the relationship between economic dependency and the risk of elder abuse, particularly among older women. A review of legal regulations and protection mechanisms in Turkey is necessary to assess the adequacy of legal processes. Research on the long‐term effects of elder abuse should identify risk factors in the aging process and contribute to the development of more effective intervention strategies. These research efforts will support the development of evidence‐based policies and protective programmes to combat elder mistreatment more effectively.

### 6.3 Implications for Practice

The study's findings emphasise the urgent need for targeted interventions to address elder abuse, particularly among older adults relying on others for daily activities. Collaboration among healthcare providers, nurses, social workers, and legislators is essential to establish comprehensive support systems, including monitoring, caregiver education, and accessible reporting mechanisms. Community‐based initiatives that enhance social support and raise awareness of elder abuse should also be implemented. Strengthening social security systems and providing financial and emotional support for caregivers are critical steps in reducing elder abuse. Culturally sensitive training programmes and interventions are needed to address the specific challenges faced by older individuals in Turkey. While the Geriatric Mistreatment Scale is effective for exploring the multidimensional aspects of elder abuse, its limitations for prevalence studies may affect the generalisability of the findings, which should be interpreted with caution.

## 7 Conclusion

This study examined the relationships between elder abuse, sociodemographic characteristics, and daily living activity levels of elderly individuals in Turkey. The results indicated that psychological abuse was the most prevalent form, while physical abuse, economic exploitation, and neglect also emerged as critical concerns. Participants frequently identified sons, spouses, and male caregivers as the primary perpetrators. Although no link was found between dependency in daily activities and abuse, sociodemographic factors such as low education, poor self‐rated health, and intrafamily conflict significantly increased the risk. Regional disparities were also prominent, with specific areas reporting markedly higher rates. These findings emphasise the urgent need for culturally sensitive assessment tools, targeted interventions, and tailored region‐specific strategies to strengthen support systems, protect vulnerable elderly individuals, and address regional disparities effectively.

## Author Contributions

All authors have agreed on the final version and meet at least one of the following criteria recommended by the ICMJE: (a) substantial contributions to conception and design, acquisition of data, or analysis and interpretation of data; (b) drafting the article or revising it critically for important intellectual content. Concept and study design: G.B.M., E.Ü. Data collection: G.B.M., E.Ü. Data analysis: G.B.M. Literature search: G.B.M., E.Ü. Manuscript writing: G.B.M. All authors supervised, critically reviewed the manuscript, and checked spelling and grammar. All the authors read and approved the final manuscript.

## Ethics Statement

The study was conducted according to the guidelines of the Declaration of Helsinki and approved by the Institutional Review Board at the Izmir Demokrasi University (Decision No: 2023/01/‐07 dated 04.01.2023).

## Consent

The written consent forms were obtained from all participants. All participants were provided with information on the study and gave consent.

## Conflicts of Interest

The authors declare no conflicts of interest.

## Data Availability

The data that support the findings of this study are available from the corresponding author upon reasonable request.
